# Transient autonomic responses during sustained attention in high and low fit young adults

**DOI:** 10.1038/srep27556

**Published:** 2016-06-08

**Authors:** Antonio Luque-Casado, Pandelis Perakakis, Luis F. Ciria, Daniel Sanabria

**Affiliations:** 1 Brain, Mind, & Behavior Research Center, University of Granada, Spain; 2 Department of Experimental Psychology, University of Granada, Spain; 3 Department of Physical Education & Sport, University of Granada, Spain; 4 Department of Personality, Evaluation & Psychological Treatment, University of Granada, Spain

## Abstract

Maintaining vigilance over long periods of time is especially critical in performing fundamental everyday activities and highly responsible professional tasks (e.g., driving, performing surgery or piloting). Here, we investigated the role of aerobic fitness as a crucial factor related to the vigilance capacity. To this end, two groups of young adult participants (high-fit and low-fit) were compared in terms of reaction time (RT) performance and event-related heart rate responses in a 60′ version of the psychomotor vigilance task. The results showed shorter RTs in high-fit participants, but only during the first 24′ of the task. Crucially, this period of improved performance was accompanied by a decelerative cardiac response pattern present only in the high-fit group that also disappeared after the first 24′. In conclusion, high aerobic fitness was related to a pattern of transient autonomic responses suggestive of an attentive preparatory state that coincided with improved behavioural performance, and that was sustained for 24′. Our findings highlight the importance of considering the role of the autonomic nervous system reactivity in the relationship between fitness and cognition in general, and sustained attention in particular.

Our ability to sustain attention for prolonged periods of time in order to respond to relevant external stimuli is far from stable. Instead, we know that an extended period of attentional demands on a single task leads to a decrement in performance over time that is usually called time-on-task effect or vigilance decrement^1^,^2^. Numerous fundamental everyday activities (e.g., attending academic lessons at school^3^ or driving^4^), and highly responsible professional tasks (e.g., performing surgery^5^, piloting^6^, or handling air-traffic control^7^) are prone to this vigilance decrement since they typically require the maintenance of task goals for long periods of time. Thus, investigation into parameters that might contribute to the capacity to sustain attention is highly relevant.

A factor that seems to positively contribute to vigilance capacity is aerobic fitness^8^. However, in spite of the extensive research on the exercise-cognition topic^9^, few studies to date have addressed the association between aerobic fitness and sustained attention^10^,^11^,^12^. Moreover, the physiological mechanisms underlying this relationship are far from clear. Here, we considered autonomic nervous system (ANS) reactivity as a key factor to shed new light into the link between sustained attention and aerobic fitness in young adults.

It is well known that regular physical activity —which results in increased physical fitness level— produces an enhanced vagal tone as a result of physiological adaptations induced by training^13^. At the same time, numerous studies have shown evidence of the close connection between the central nervous system (CNS) and the ANS^14^. Both the CNS and the ANS are reciprocally interconnected and maintain a bidirectional relationship. As a clear example of this, there are numerous studies showing the role of vagally-mediated heart rate variability (HRV; used as an index of the ANS control of the cardiovascular system^15^), in the regulation of physiological, affective, and cognitive processes^14^,^16^. It is suggested that individuals with higher resting HRV (indicating an efficient vagal control on the ANS functioning) are better able to perform tasks that involve executive and inhibitory functions^17^,^18^ or sustained attention^19^. In addition, certain studies related the increased vagally-mediated ANS functioning in high-fit individuals (as a result of chronic exercise) to better cognitive performance in executive tasks^20^,^21^. Taken together, the available evidence suggests that ANS functioning could be a key physiological mechanism involved in the improved ability to sustain attention in high-fit individuals.

To the best of our knowledge, only the study by Luque-Casado, Zabala, Morales, Mateo-March, and Sanabria^8^ has directly investigated the association between ANS functioning and sustained attention performance as a function of aerobic fitness. This study showed better vigilance performance in a 10′ version of the psychomotor vigilance task (PVT) indexed by shorter overall reaction times (RTs) in high-fit than in low-fit young adults. Additionally, a linear decrement in HRV as a function of time-on-task was shown only in low-fit participants, with HRV remaining steady during task performance in the high-fit group. These results were taken as evidence of superior sustained attention capacity that was accompanied by an improved functioning of cardiac autonomic control in high-fit compared to low-fit individuals.

The use of HRV parameters indexing parasympathetic tone does not allow the direct investigation of a possible relationship between task performance and ANS reactivity on a trial-by-trial basis. To elucidate this relationship, here we assessed heart rate responses evoked by the preparatory cue stimulus of the PVT to examine autonomic reactivity. Evoked heart rate responses, also known as Phasic Cardiac Responses (PCRs), have often been explored using the paired stimulus (S1–S2) paradigm, in which a tri-phasic heart rate response between warning (S1) and imperative stimuli (S2) has been described^22^. First, an initial brief heart rate deceleration occurs after the warning onset, followed by heart rate acceleration over the next several cardiac beats. Subsequently, a new sustained heart rate deceleration occurs prior to the imperative stimuli. Although the phasic cardiac changes found in the S1–S2 inter-stimulus interval have been shown to be vagally-mediated^23^, the parasympathetic nerve traffic enacts its effects at a much faster rate (<1 s) than sympathetic outflow (2.5 s for influencing heart rate and 5 s in influencing peripheral resistance)^24^,^25^,^26^. Thus, by selecting short latency epochs (i.e., 1 s) after the stimulus onset, it is highly likely that the measured heart rate responses are determined primarily by discharges of the vagus nerve. Consequently, this cardiac autonomic marker is an excellent index to capture any between-groups differences mediated by vagal reactivity.

To further explore whether the improved performance of high-fit young adults observed in our previous report is stable or decays as a function of task duration, which is a fundamental feature taxing sustained attention^1^, in this study we employed for the first time a long-duration 60′ version of the PVT.

## Method

### Participants

A minimum sample size of 22 participants per group was required for a power level of 0.80 as determined by an *a priori* power analysis based on data from a previous study^8^. Thus, fifty young adult participants (twenty-five per group) were recruited from a larger pool of eighty-nine undergraduate students from the University of Granada and members of local triathlon clubs. The participants in the high and low-fit group met the inclusion criteria of reporting at least 8 hours of training per week or less than 2 hours, respectively. Six of the fifty participants were subsequently excluded from the analyses (see data reduction section). Descriptive data from the remaining 44 participants are reported in Table 1.

The study methods were carried out in accordance with the Declaration of Helsinki 1964 and all experimental protocols and procedures were approved by the ethical committee of the University of Granada. All participants were informed about their right to interrupt the experiment at any moment and gave informed consent prior to their inclusion in the study. They were required to maintain a regular sleep–wake cycle for at least one day before the study and to avoid caffeine and vigorous physical activity before the visit to the laboratory. All participants’ data were analysed and reported anonymously.

### Procedure

All participants received verbal and written information about the experiment upon their arrival to the laboratory. They were seated in front of a computer in a dimly-illuminated, sound-attenuated Faraday room and were prepared for electrophysiological measurement. These preliminary steps lasted around 15 minutes in which the subjects rested in a sitting position, thereby preventing any alteration in the subsequent baseline recording. Initial baseline electrocardiogram (ECG) signal was recorded for 5 minutes in a sitting position. The participants were encouraged to stay as relaxed as possible during this time period. Then, they received verbal and written instructions regarding the PVT and practiced for one minute before completing a 60′ version of the task. ECG signal was continuously recorded during the experiment. Subsequently, all participants performed a submaximal cardiorespiratory fitness test to evaluate their fitness level. This test was performed after the PVT in order to avoid the influence of physical effort on cognitive performance^27^. The entire experimental session lasted 2 h approximately.

### The Psychomotor Vigilance Task

We used a PC with a 19″ monitor and E-Prime software (Psychology Software Tools, Pittsburgh, PA, USA) to control stimulus presentation, response collection, and to generate and send triggers indicating the condition of each trial for offline sorting, reduction, and analysis of ECG and behavioural data. The centre of the PC screen was situated ~60 cm from the participant’s head and at eye level. The device used to collect responses was a PC keyboard.

The procedure of the PVT was based on the original version^28^. This task was designed to measure vigilance by recording participants’ RT to visual stimuli that occur at random inter-stimulus intervals^28^,^29^. Each trial began with the presentation of a blank screen in a black background for 2000 ms and subsequently, an empty red circumference (i.e., cue stimulus, 6.68° × 7.82° of visual angle at a viewing distance of 60 cm) appeared in a black background. Later, in a random time interval (between 2000 and 10000 ms), the circumference was filled all at once in a red colour (i.e., target stimulus). Participants were instructed to respond as fast as they could once they had detected the presentation of the filled circle. The filled circle was presented for 500 ms and the participants had a maximum of 1500 ms to respond. They had to respond with their dominant hand by pressing the space bar on the keyboard. A RT visual feedback message was displayed for 300 ms after response, except in case of an anticipated response (“wait for the target”) or if no response was made within 1000 ms after target offset (“you did not answer”). Following the feedback message the next trial began. Response anticipations were considered errors. The task comprised a single block of 60 minutes of total duration and the mean number of trials per participant was 415 ± 6.3.

### Submaximal cardiorespiratory fitness test

Prior to the start of the fitness test, descriptive anthropometric parameters of weight, height and body mass index (BMI) were obtained for each participant (see Table 1). Then, all participants were fitted with a Polar RS800 CX monitor (Polar Electro Öy, Kempele, Finland) to record their heart rate (HR) during the incremental exercise test. We used a ViaSprint 150 P cycle ergometer (Ergoline GmbH, Germany) to induce physical effort and to obtain power values and a JAEGER Master Screen gas analyser (CareFusion GmbH, Germany) to provide a measure of gas exchange during the test.

The incremental effort test started with a 3 minutes warm-up at 30 Watts (W), with the power output increasing 10 W every minute. During this warm-up period, each participant set his preferred cadence (between 60–90 rev · min^−1^) and was asked to maintain this cadence throughout the protocol. The test began at 60 W and was followed by an incremental protocol with the power load increasing 30 W every 3 minutes. Workload increased progressively during the third minute of each step (5 W every 10 seconds [s]); therefore, each step of the incremental protocol consisted of 2 minutes of stabilized load and 1 minute of progressive load increase. The oxygen uptake (VO2 ml·min^−1^·kg^−1^), respiratory exchange ratio (RER; i.e., CO_2_ production·O_2_ consumption^−1^), relative load (W·Kg^−1^), heart rate (bpm) and time of the test (s) were continuously recorded during the entire incremental test.

We used the ventilatory anaerobic threshold (VAT) as a reference to determine the fitness level of the participants (see Table 1). VAT is considered to be a sensitive measure for evaluating aerobic fitness and cardiorespiratory endurance performance^30^,^31^ and was defined as the VO_2_ at the power load in which RER exceeded the cut-off value of 1.0^32^,^33^. The researcher knew that the participant had reached his VAT when the RER was equal to 1.00 and did not drop below that level during the 2 minutes constant load period or during the next load step, never reaching the 1.1 RER. The submaximal cardiorespiratory fitness test ended once the VAT was reached.

### Electrocardiogram (ECG) recordings

Continuous ECG data were acquired using a BioSemi Active Two amplifier system (Biosemi, Amsterdam, Netherlands). The signal was digitised at a sampling rate of 1024 Hz with 24-bit A/D conversion. Two FLAT active electrodes (Ag/AgCl; Biosemi, Amsterdam, Netherlands) were arranged at a modified lead I configuration (i.e., right and left wrists). Before attaching the electrodes to the participant, electrode sites for the measurement of the ECG were prepared by cleaning the skin with ethyl alcohol (70%). Signa Electro-Gel (Parker Laboratories, Fairfield, NJ, USA) was used to optimize the electrodes signal transduction. The signal was visualised on a computer screen to check for good electrode contact before starting the data acquisition. Participants were instructed to avoid body movements as much as possible during the experiment.

### Data reduction

The behavioural data analyses were performed on the overall participants’ mean RTs. Trials with RTs below 100 ms (0.03%), anticipations (i.e., responses prior to the target presentation; 1.34%) and omissions (if no response was made within 1000 ms after target offset; 0.20%) were discarded from the analyses^29^.

Continuous ECG raw data were filtered offline using a band-pass 0.5–50 Hz filter. R-wave detection and artefact correction were performed with the ECGLab Matlab software^34^. Six participants (i.e., two high-fit and four low-fit) were excluded from further analyses due to poor signal quality. We used the KARDIA Matlab software^35^ and bespoke Matlab scripts (Matlab 2013a, Mathworks Inc.) to analyse the heart period signal at baseline and during the execution of the PVT. The average inter-beat interval (IBI), the root-mean-square difference of successive normal R-R intervals (rMSSD) and the absolute power of the high-frequency spectral component (HF ms^2^; [0.15 to 0.40 Hz]) were obtained during a 5-minute baseline period as indexes of resting vagal tone^15^ (see Table 1).

To assess the PCR to the cue stimulus (i.e., the empty circumference) in a single trial, we first calculated the weighted average heart period for a time window of 1 s following cue onset, using the fractional counting procedure^36^. We subsequently subtracted the weighted average heart period calculated for a window 0.5 s before the cue onset, in order to obtain heart period changes with respect to baseline activity. Group average PCRs were obtained by averaging across trials and subjects. Trials including questionable IBIs (0.49%) or trials that did not meet the criteria set for behavioural analyses (1.57%), were rejected and not used in data averages. A minimum of 53 trials per condition was maintained.

### Design and Statistical analysis

Three sets of dependent variables were evaluated in this study: 1) Participants’ descriptive and fitness data parameters (i.e., anthropometrical, average IBI, rMSSD, HF ms^2^, and incremental exercise test); 2) behavioural data (i.e., overall mean RTs); and 3) PCR data during the PVT. Five temporal blocks of 12 minutes were considered to measure the time-on-task effect on the behavioural and PCR data. Nonparametric permutation tests were used for statistical analysis^37^,^38^. We followed a general label exchange procedure for within-participants factorial designs^39^ using a Monte Carlo approach.

The participants’ descriptive and fitness data were analysed using 1-way between-groups design. For the behavioural and PCR data, we had a factorial design with the between-groups variable of group (high-fit and low-fit) and the within-groups variable of time-on-task (block1, block2, block3, block4 and block5). Significant main effects and interactions were further explored by using pairwise comparisons. Multiple comparisons correction was accounted for by applying the false discovery rate (FDR) approach. 95% confidence intervals (CI) and probability threshold values are reported.

## Results

### Descriptive and fitness data

The permutations tests showed significant differences between groups in the average IBI, rMSSD, and all the fitness test parameters (i.e., time to VAT (s), relative power output (W·kg^−1^) at VAT and VO_2_ (mL·min^−1^·kg^−1^) at VAT) (all *ps* ≤ 0.01). All data evidenced the difference in fitness level between groups (see Table 1). No statistically significant differences between groups were shown in HF absolute power at baseline (*p* = 0.14) nor in any of the anthropometrical parameters (all *ps* ≥ 0.08).

### Behavioural results

Participants’ mean RTs results showed significant main effects of group (*p* < 0.01) and time-on-task (*p* < 0.01). Crucially, the interaction between both factors also reached statistical significance (*p* < 0.01; see Table 2). Pairwise comparisons between groups were performed within each temporal block (FDR corrected; *p*-threshold = 0.009). The comparisons showed significant differences between groups at block 1 and 2 (both *ps* < 0.001) with high-fit being faster than low-fit group. There were no significant differences when comparing groups at block 3 and 4 (both *ps* ≥ 0.18). The between groups differences reached again statistical significance (*p* = 0.009) at block 5, but with the high-fit being slower than the low-fit group in this case (see Table 2).

### PCR results

The PCR analyses revealed significant main effects of group (*p* < 0.01) and time-on-task (*p* < 0.01). Again, the interaction between group and time-on-task reached statistical significance (*p* < 0.01; see Table 2). Pairwise comparisons (FDR corrected; *p-*threshold = 0.0001) showed significant differences between groups at blocks 1 and 2 (both *ps* ≤ 0.0001). In both cases, the high-fit group showed greater cardiac deceleration than the low-fit group (see Table 2). There were no significant differences between groups in the remaining blocks (all *ps* ≥ 0.04).

## Discussion

We examined the relationship between aerobic fitness, behavioural performance and autonomic reactivity of young adults in a prolonged sustained attention task. To this end, two groups of participants (i.e., high-fit and low-fit) were compared in terms of RT performance and PCRs elicited by stimulus presentation over time in a 60′ version of the PVT.

High-fit individuals showed greater resting vagal control than low-fit presumably as a result of physiological adaptations induced by training^13^. Therefore, according to previous research^8^,^19^, one could have expected better overall performance of the high-fit group with respect to the low-fit group in the PVT. The behavioural results reported here are partially in agreement with this previous evidence. Indeed, high-fit individuals showed shorter RTs than low-fit in the first two blocks of the task (i.e., 24 min), but this improved performance disappeared as a function of time-on-task. This is a novel finding since no previous study reported differences in the fitness-related improved performance as a function of task duration in young adults^8^,^12^. Here, it is important to note that these previous studies used experimental tasks that typically last for only a few minutes (i.e., 10 minutes at the most), a duration that might be insufficient to elicit a significant deterioration in vigilance performance in young adults^2^. Thus, the duration of the task appears to be a key factor to pinpoint the link between aerobic fitness and sustained attention.

The PCR results showed a decelerative response pattern only for the high-fit group. Because PCR magnitude is positively related to vagal tone^23^, it is plausible that the enhanced vagal control in high-fit promoted greater autonomic flexibility, facilitating in turn the observed event-related cardiac decelerations. On the other hand, the absence of heart rate decelerations in low-fit participants suggests a pervasive lack of parasympathetic modulation of heart rate not permitting a vagally-mediated dynamic response to transient stimuli. In fact, sedentary lifestyle is linked to autonomic imbalance and decreased parasympathetic tone^40^,^41^, which has been related to behavioural dynamic inflexibility and inefficient attentional regulation^42^.

Interestingly, the phasic decelerative cardiac responses were observed in high-fit individuals only during the first two blocks of the task, coinciding in time with their improved RT performance relative to their low-fit counterparts. This temporal correspondence suggests an association between the transient cue-related vagal discharges and the performance in the attention task. Given that participants were instructed to focus and maintain attention to the cue stimulus (to be ready for a fast response to the target), it could be argued that the event-related cardiac decelerations shown by high-fit individuals in our study were induced by a greater attentive preparatory state. Indeed, a cardiac deceleration in response to stimulus presentation has been related to attentional processing^43^, with greater magnitudes under conditions of high uncertainty and high vigilance demands^44^.

Maintaining a high attentional state is effortful and it is taxed by time-on-task^2^,^45^. This could explain why high-fit participants showed a greater capacity to deploy attention only for the first 24′ of the task. Alternatively, a habituation effect (i.e., a gradual familiarity effect and reduction of the cue-related heart response with repeated stimulus presentation), could also have been responsible of the disappearance of the observed cardiac deceleration in higher-fit individuals^43^,^46^,^47^. However, this habituation process usually occurs within a range of few trials^43^, while, in our study, the attenuation of cardiac deceleration appeared after two blocks of the task (i.e., 24 minutes). Therefore, although this possibility cannot be completely ruled out, it would seem unlikely that mere habituation was responsible of the disappearance of the event-related heart deceleration. In any case, high-fit participants showed greater vagally-mediated cardiac responses than low-fit that were related to improved RT performance in the PVT, whatever the mechanism responsible of the attenuated heart response after the first half of the task.

In summary, higher aerobic fitness was related to stronger transient autonomic responses and improved behavioural performance indicative of an enhanced attentive preparatory state that was maintained only during the first part of the task. In general terms, the current dataset replicates and extends this area of research by demonstrating, for the first time, an association between aerobic fitness, ANS reactivity, and sustained attention. Our findings are highly relevant to the topic of fitness and cognition and advice of the importance of considering the role of the ANS functioning in the relationship between fitness and cognition in general, and attentional performance in particular. Hence, future research would benefit from study designs that combine measurements of ANS and brain functioning, which would make possible to gain more insight into the physiological mechanisms involved in fitness-related improvements in cognition.

## Additional Information

**How to cite this article**: Luque-Casado, A. *et al.* Transient autonomic responses during sustained attention in high and low fit young adults. *Sci. Rep.* **6**, 27556; doi: 10.1038/srep27556 (2016).

## Figures and Tables

**Table 1:** Mean and 95% Confidence Interval (CI) of descriptive and fitness data for the high-fit and low-fit groups.

	High-fit	Low-fit
Anthropometrical characteristics		
Sample size^a^	23	21
Age (years)	23 [21, 24]	23 [22, 24]
Height (cm)	1.77 [1.75, 1.79]	1.78 [1.75, 1.81]
Weight (kg)	69.4 [67.0, 71.9]	77.3 [69.4, 85.9]
Body Mass Index (kg·(m^2^)^−1^)	22.2 [21.5, 22.9]	24.2 [22.2, 26.3]
Baseline cardiac parameters		
Average IBI (ms)	1005.1 [931.9, 1078.7]	758.0 [719.3, 799.1]
rMSSD (ms)	59.7 [45.0, 76.2]	35.9 [28.3, 44.1]
HF (ms^2^)	1749.1 [918.4, 2843.3]	885.6 [526.5, 1319.9]
Fitness test parameters		
Time to VAT (s)	1291 [1186.7, 1392.8]	471 [407.4, 536.1]
VO_2_ (mL·min^−1^·kg^−1^) at VAT	43.8 [40.6, 47.2]	18.8 [16.7, 21.0]
Relative power output at VAT (W·kg^−1^)	3.46 [3.16, 3.77]	1.34 [1.18, 1.51]

^a^Only data of the participants included in the analyses are reported; IBI = inter-beat interval; rMSSD = the root-mean-square difference of successive normal R-R intervals; HF (ms^2^) = absolute power of the high-frequency spectral component (0.15 to 0.40 Hz); VAT = ventilatory anaerobic threshold.

**Table 2:** Mean and 95% Confidence Interval (CI) for the behavioural and phasic cardiac responses (PCRs) data as a function of Group and Block.

	Block 1	Block 2	Block 3	Block 4	Block 5
High-fit					
RTs (ms)	259.8 [257.6, 262.0]	274.2 [271.9, 276.5]	282.1 [279.2, 285.1]	294.7 [291.6, 298.0]	302.2 [298.9, 305.5]
PCRs (ms)^a^	2.27 [0.19, 4.47]	2.78 [0.42, 5.08]	−1.57 [−4.15, 1.14]	−4.05 [−6.70, −1.29]	−6.56 [−9.23, −3.81]
Low-fit					
RTs (ms)	273.1 [270.4, 276.0]	282.5 [279.4, 285.6]	285.1 [282.0, 288.4]	292.9 [289.0, 296.9]	295.6 [292.0, 299.3]
PCRs (ms)^a^	−3.20 [−4.63, −1.71]	−4.47 [−5.85, −2.98]	−4.78 [−6.11, −3.43]	−4.64 [−6.28, −3.01]	−5.83 [−7.52, −4.10]

^a^Weighted heart period (ms) for 1 s epochs relative to cue onset and baseline-corrected taking 0.5 s pre-stimulus onset into account. Block1 (0–12 minutes); Block2 (12–24 minutes); Block3 (24–36 minutes); Block4 (36–48 minutes); Block5 (48–60 minutes).
